# Selection of Diagnostic Cutoffs for Murine Typhus IgM and IgG Immunofluorescence Assay: A Systematic Review

**DOI:** 10.4269/ajtmh.19-0818

**Published:** 2020-04-06

**Authors:** Sandhya Dhawan, Matthew T. Robinson, John Stenos, Stephen R. Graves, Tri Wangrangsimakul, Paul N. Newton, Nicholas P. J. Day, Stuart D. Blacksell

**Affiliations:** 1 Mahidol Oxford Tropical Medicine Research Unit, Faculty of Tropical Medicine, Mahidol University, Bangkok, Thailand;; 2 Centre for Tropical Medicine and Global Health, Nuffield Department of Medicine, University of Oxford, Oxford, United Kingdom;; 3 Lao-Oxford-Mahosot Hospital-Wellcome Trust Research Unit (LOMWRU), Mahosot Hospital, Vientiane, Lao People’s Democratic Republic;; 4 Australian Rickettsial Reference Laboratory, University Hospital Geelong, Geelong, Australia

## Abstract

Murine typhus is a neglected but widespread infectious disease that results in acute fever. The immunofluorescence assay (IFA) is the “gold standard” to identify IgM or IgG antibodies, although there is a lack of standardization in methodologies. The objective of this review is to summarize 1) the differences in published methodologies, 2) the diagnostic cutoff titers, and 3) the justification of diagnostic cutoffs. Searches were performed by combining the following search terms: “murine typhus,” “*rickettsia typhi*,” “immunofluorescence,” “IFA,” and “serologic” with restrictions (i.e., “*rickettsia typhi*” or “murine typhus,” and “IFA” or “immunofluorescence,” or “serologic*”). The search identified 78 studies that used IFA or immunoperoxidase assay (IIP) antibody cutoffs to diagnose murine typhus, 39 of which were case series. Overall, 45 studies (57.7%) provided little to no rationale as to how the cutoff was derived. Variation was seen locally in the cutoff titers used, but a 4-fold or greater increase was often applied. The cutoffs varied depending on the antibody target. No consensus was observed in establishing a cutoff, or for a single-value diagnostic cutoff. In conclusion, there is a lack of consensus in the establishment of a single-value cutoff. Further studies will need to be executed at each distinct geographic location to identify region-specific cutoffs, while also considering background antibody levels to distinguish between healthy and infected patients.

## INTRODUCTION

Murine typhus is a neglected infectious disease caused by *Rickettsia typhi*, a Gram-negative, obligate intracellular bacterium. *Rickettsia typhi* is primarily transmitted by *Xenopsylla cheopis*, the rat flea.^1^ Commensal rats (most commonly *Rattus rattus* and *Rattus norvegicus*) are the natural animal reservoir of the disease. Infection in humans occurs either through inoculation of infected flea feces into bite wounds or by inhalation of aerosolized flea feces.^2–4^

Given that other febrile illnesses, such as dengue, leptospirosis, and typhoid, have similar clinical manifestations to murine typhus,^3,5^ laboratory tests are essential to differentiate murine typhus from other causes of undifferentiated fever. Serological methods are commonly used to diagnose murine typhus because of their simplicity and cost-effectiveness.^6,7^ The indirect immunofluorescence assay (IFA) is considered the “gold standard” and reference technique for diagnosing murine typhus in most research laboratories.^1–3,8^ Immunofluorescence assay identification of IgM and IgG antibodies provides definitive and accurate evidence of exposure.^2,9,10^ The immunoperoxidase assay (IIP) is an alternative to IFA and obtains results that have a similar sensitivity and specificity.^11^

The diagnostic accuracy of IFA is subjective and reliant on methodological and patient factors. Despite being the current reference and standard technique, there is little consensus on the standardization of the IFA methodology. Variable methodological factors include the antigenic strains used and antibody isotype targeted, as well as the diagnostic cutoffs used. Therefore, to guarantee accuracy of diagnosis, standardized methodologies and locally authenticated positivity cutoff limits for diagnostic and epidemiological purposes are required.

This review aims to summarize 1) the differences in published IFA methodologies, 2) the diagnostic cutoff titers used for a positive murine typhus diagnosis, and 3) the justification of these diagnostic cutoffs.

## METHODS

### Search strategy and eligibility criteria.

A systematic review was performed. Searches were performed by one author (S. D.) on the PubMed electronic database by combining the following search terms: “murine typhus,” “*rickettsia typhi*,” “immunofluorescence,” “IFA,” and “serologic” with restrictions (i.e., “*rickettsia typhi*” or “murine typhus,” and “IFA” or “immunofluorescence,” or “serologic*”). The search was limited to articles that had been published in or could be successfully translated to English, until July 2018. First, the titles and abstracts were screened for applicability. Then, full text of relevant articles were examined to establish eligibility. Diagnostic accuracy studies, case series, and cross-sectional studies using IFA/IIP to diagnose murine typhus were included. We excluded case reports, nonhuman studies, and studies investigating other serological tests (i.e., CF, OX-19, and ELISA). Reference lists of the selected studies were also screened to identify further studies.

### Data extraction and analysis.

Data were extracted by one author (S. D.), and where the information was unclear, a second researcher was consulted (S. D. B.). Details of the location, sample size, study design, reference test, positivity cutoff titer, antibody target, antigenic strain, positivity criteria, and justification for positive cutoff titer were compiled into summary tables. The studies were grouped according to the study design (diagnostic accuracy study, case series, or cross-sectional study) and geographical location. The data were summarized using a narrative synthesis. We did not evaluate intricacies of individual IFA protocols but instead focused on the broader issues such as the methodology used to derive diagnostic cutoffs.

## RESULTS

### Summary of studies.

#### Study types.

Of the total of 78 studies included in this review (Table 3), 39 (49.4%) were case series, 34 (43%) were cross-sectional studies, and five (6.33%) were diagnostic accuracy studies (Supplemental Table 3, Tables 1 and 2).

**Table 1:** Summary of cross-sectional studies

Country	Type of test	Source of assay	Total cases	Antigenic strain	Positivity cutoff titer	Antibody target	Positivity criteria	Cutoff justification	Reference
American Samoa	IFA	NA	197	NA	1:50	IgG	Single titer	NA	44
Brazil	IFA	NA	437	NA	> 1:64	IgM	Both	NA	45
					≥ 4-fold increase	IgM			
					1:64	IgG	Single titer		
Croatia	IFA	Virus Reference Laboratory, London, UK	425	NA	≥ 1:16	IgG	Single titer	NA	46
Djibouti	IFA	NA	12,300	NA	≥ 1:80	NA	Both	36	47
					≥ 4-fold increase	NA			
Greece	IFA	Biomerieux, Marcy l'Etoile, Lyon, France	1,584	NA	≥ 1:64	IgG	Single titer	NA	48
Indonesia	IFA	NA	142	NA	≥ 1:80	NA	Single titer	32,35	49
Lao PDR	IFA	NA	427	NA	> 1:64	IgM	Both	6,31	50
					≥ 4-fold increase	IgM and IgG			
					> 1:128	IgG			
Madagascar	IFA	NA	31	NA	1:4000	IgG	Single titer	NA	51
Malaysia	IFA	NA	1596	Wilmington	≥ 1:50	Whole	Single titer	52	53
Morocco	IFA	Biomerieux, Marcy l’Etoile, Lyon, France	300	Moroccan strain	≥ 1:32	NA	Single titer	20,21	15
Nepal	IFA	NA	103	Wilmington	≥ 1:400	IgM	Both	17	54
					≥ 4-fold increase	IgM			
New Zealand	IFA	Australian Rickettsial Reference Laboratory, Victoria, Australia	989	NA	≥ 1:128	IgG	Single titer	Manufacturer’s specifications	39
Singapore	IIP	U.S. Army Medical Research Unit, Malaysia	35	NA	≥ 1:1600	IgG	Both	Manufacturer’s specifications	38
					≥ 1:400	IgG			
					≥ 4-fold increase	IgG			
Spain	IFA	NA	341	NA	1:40 – 1:160	NA	Range	NA	55
	IFA	Biomerieux, Marcy l'Etoile, Lyon, France	662	NA	≥ 1:80	IgG	Single titer	Manufacturer’s specifications	37
	IFA	Focus technologies, Cypress, CA	734	NA	≥ 1:64	IgM	Both	NA	56
					≥ 4-fold increase	IgM			
					≥ 1:64	NA			
	IFA	NA	104	NA	≥ 1:512	IgG	Both	NA	57
					≥ 4-fold increase	IgG			
	IFA	NA	640	Wilmington	≥ 1:128	IgG	Single titer	NA	14
					≥ 1:40	IgM, IgG, IgA	Single titer		
	IFA	MRL Diagnostics, Cypress, CA	217	NA	≥ 1:40	IgG	Single titer	NA	58
	IFA	NA	400	NA	1:40	NA	Single titer	NA	59
	IFA	Focus technologies, Cypress, CA	504	ref no. IF0100	≥ 1:64	NA	Single titer	NA	60
	IFA	NA	383	Wilmington	≥ 1:80	IgG	Single titer	NA	61
	IFA	Biomerieux, Marcy l'Etoile, Lyon, France	356,266	NA	≥ 1:40	NA	Single titer	NA	62
Taiwan	IFA	Taiwan CDC, Taipei, Taiwan	226	NA	≥ 1:80	IgM	Single titer	NA	63
					≥ 4-fold increase	IgG	Only 4-fold		
	IFA	Taiwan CDC, Taipei, Taiwan	1420	NA	≥ 4-fold increase	NA	Only 4-fold	NA	64
Tanzania	IFA	NA	870	Wilmington	≥ 1:64	IgM	Single titer	NA	65
					≥ 4-fold increase	IgG	Only 4-fold		
	IFA	NA	150	ATCC AZ332	> 1:50	Whole	Single titer	21	66
Tunisia	IFA	NA	500	NA	≥ 1:32	Whole	Single titer	20	67
	IFA	NA	47	NA	≥ 1:32	IgM	Single titer	NA	68
	IFA	NA	1024	NA	> 1:128	IgG	Single titer	NA	69
					≥ 1:32	IgM	Single titer		
United States	IFA	NA	204	NA	≥ 1:64	IgG	Single titer	NA	70
	IFA	Focus technologies, Cypress, CA	152	NA	≥ 1:64	IgG	Single titer	NA	71
Vietnam	IFA	In-house	193	Wilmington	1:400	IgG	Both	54	72
					≥ 4-fold increase	IgG			
Zambia	IFA	NA	377	Wilmington	≥ 1:32	IgM	Single titer	NA	73
					≥ 1:64	Whole	Single titer		

IFA = immunofluorescence assay; IIP = immunoperoxidase assay.

**Table 2:** Summary of diagnostic accuracy studies

Country	Type of test	Source of assay	Total cases	Antigenic strain	Positivity cutoff titer	Antibody target	Positivity criteria	Cutoff justification	Reference
Israel	IFA	NA	23	NA	≥ 1:100	Whole	Single titer	NA	74
Lao PDR	IFA	Australian Rickettsial Reference Laboratory, Victoria, Australia	1030	Wilmington	≥ 1:400	IgM	Single titer	17	75
					≥ 4-fold increase	IgM and IgG	Only 4-fold		
	IFA	NA	50	Wilmington	≥ 1:400	Whole	Both	17	76
					≥ 4-fold increase	Whole			
Peru, United States, Somalia, and Indonesia	IFA	NA	60	Wilmington	≥ 1:128	IgG	Single titer	NA	8
Russia, Peru, and Burundi	IFA	Dynatech Laboratories Ltd, UK	308	Wilmington	≥ 1:128	IgG	Single titer	25,26	13

IFA = immunofluorescence assay.

#### Patient and geographic details.

The study year of included articles ranged from 1977 to 2018. The total number of cases analyzed was 392,756. Geographically, the studies were conducted on patients from Spain (12.8%, *n* = 10), Taiwan (9.0%, *n* = 7), United States (7.7%, *n* = 6), Lao PDR (6.4%, *n* = 5), Tunisia (6.4%, *n* = 5), Thailand (6.4%, *n* = 5), and Greece (6.4%, *n* = 5). The remaining study populations were recruited from American Samoa, Australia, Brazil, China, Colombia, Croatia, Cyprus, Djibouti, France, Germany, Indonesia, Israel, Madagascar, Malaysia, Malta, Morocco, Nepal, New Zealand, Singapore, Sri Lanka, Tanzania, Vietnam, and Zambia (Table 3). One study conducted in Marseilles, France, investigated travelers returning from Africa and Southeast Asia.^12^ Two studies examined serum samples from three different countries.^8,13^

**Table 3:** Summary of cutoff titer positivity criteria and antibody isotype described in selected studies

	Positivity cutoff titer criteria					Antibody target (n studies)					
	Single titer	Both	Only 4-fold	Range	Total	IgM	IgG	Whole	IgM/IgG	Not stated	Isotype total
Country											
American Samoa	1				1		1				1
Australia	1				1				1		1
Brazil	1	1			2				1		1
China	2				2				1		1
Colombia	1		1		2				1		1
Croatia	1		1		2		1	1			2
Cyprus	4				4				2		2
Djibouti		1			1					1	1
France	3				3				1	1	2
Germany	1				1		1				1
Greece	5	3			8	1	1		2	1	5
Indonesia	1	3			4				1	1	2
Israel	2				2	1		1			2
Lao PDR	1	2	2		5			3	2		5
Madagascar	1				1		1				1
Malaysia	1				1		1	1			2
Malta	1				1	1					1
Morocco	1				1					1	1
Nepal	1	1	1		3	1			1		2
New Zealand	1				1		1				1
Singapore		1			1		1				1
Spain	8	2		1	11	1	4			4	9
Sri Lanka	1	3		1	5	1			1		2
Taiwan	6		7		13				6	1	7
Tanzania	2		1		3			1	1		2
Thailand		5			5			1		4	5
Tunisia	6	3			9	2		1	2	1	6
United States	2	4			6		3		1	2	6
Vietnam		1			1		1				1
Zambia	2				2			1			1
**Total**	**57**	**30**	**13**	**2**	102*	**8**	**16**	**10**	**24**	**17**	**75****†**
Study design											
Case series	21	13	4	1	39	6	3	4	17	9	39
Cross-sectional studies	24	8	1	1	34	3	13	4	6	8	34
Diagnostic accuracy studies	4	1			5		2	2	1		5
** Total**	**49**	**22**	**5**	**2**	**78**	**9**	**18**	**10**	**24**	**17**	**78**

* Some studies provided different positivity criteria for IgM and IgG.

† Three studies were not included as they examined murine typhus in travelers from various countries.

### Immunofluorescence assay methodology.

#### Source.

More than half of the studies did not specify the source of the IFA kits (57.7%, *n* = 45). Thirty-two studies (41%) specified the source of the IFA kits, of which BioMérieux (BioMérieux Ltd., Marcy-l’Étoile, Lyon, France) was the most common source used in nine studies (27.3%, 9/33). Five studies (15.2%, 5/33) used IFA methods developed by the Australian Rickettsial Reference Laboratory (ARRL), whereas five used IFA methods developed by the U.S. Army Medical Research Unit, Malaysia.

#### Antibody isotype.

Of the 78 studies evaluated, 61 stated the target antibody isotype, whereas 17 studies (21.8%, 17/78) did not specify the antibody isotype being targeted. The majority of the studies tested for both IgM and IgG (37.7%, 23/61) against *R. typhi*. Eighteen studies (29.5%, 18/61) tested exclusively for IgG, whereas nine studies (14.8%, 9/61) tested solely for IgM. Ten studies (16.4%, 10/61) performed whole antibody testing (both IgM and IgG). In one case (1.6%, 1/61), IgM, IgG, and IgA were tested for.^14^

#### Antigenic composition.

A narrow range of antigens were used in the IFAs examined. More than half of the studies did not specify the antigenic strain used (67.9%, *n* = 53); of the 24 studies that did, the Wilmington strain was the most numerous—in 21 studies (87.5%, 21/24). Of the nine studies using BioMérieux IFAs, eight studies (88.9%, 8/9) did not specify the antigenic strain used, whereas one (11.1%, 1/9) used the Moroccan strain.^15^ Five studies used ARRL developed IFAs, of which 3 (60%, 3/5) used the Wilmington strain and two (40%, 2/5) did not specify the antigenic strain used. Five studies used IFAs developed by the U.S. Army Medical Research Unit, Malaysia, of which two (40%, 2/5) used the Wilmington strain and three (60%, 3/5) did not specify the strain used.

### Cutoffs used and methodology for selecting cutoffs.

#### Diagnostic cutoffs.

**Figure 1.**
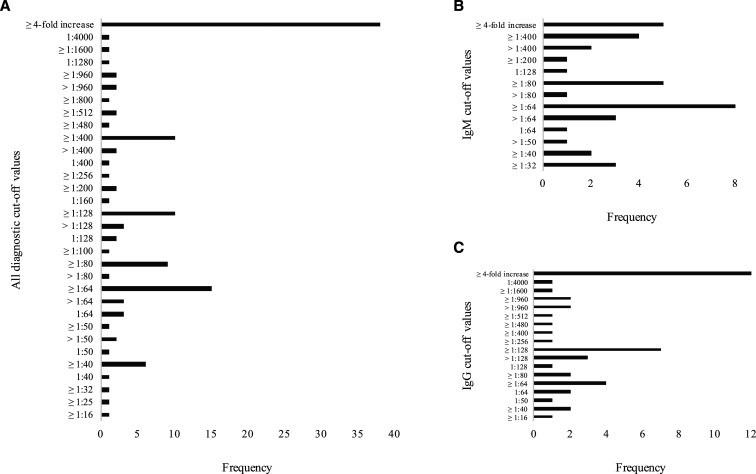
Diagnostic cutoff values’ frequency. (**A**) The diagnostic cutoff values observed in all the studies included in this review were compiled into this chart. The frequency of each cutoff value is shown. The lowest frequency observed was 1; the highest frequency observed was 38. Two studies that described a range of cutoffs were omitted. (**B**) The diagnostic cutoff values observed for the IgM isotype were compiled. The lowest frequency observed was 1; the highest frequency observed was 8. Two studies that used a range of cutoffs were omitted. (**C**) The diagnostic cutoff values observed for the IgG isotype were compiled. The lowest frequency observed was 1; the highest frequency observed was 12.

All studies show considerable variation between the cutoffs (Figure 1). Diagnostic cutoffs for IgM ranged from ≥ 1:32 to ≥ 1:400, and IgG cutoffs ranged from ≥ 1:16 to ≥ 1:960 (Figure 1B and C). From the 78 studies included, the most common cutoffs noted for IgM were ≥ 1:64 (10.2%, *n* = 8), followed by a ≥ 4-fold increase (6.4%, *n* = 5) in paired samples, and ≥ 1:80 (6.4%, *n* = 5) (Figure 1B). The most common cutoffs noted for IgG were a ≥ 4-fold increase (15.4%, *n* = 12) in paired samples, followed by ≥ 1:128 (9.0%, *n* = 7), and ≥ 1:64 (5.1%, *n* = 4) (Figure 1C). Of these studies, 23 (29.5%, 23/78) stated cutoffs for IgG and IgM. Eighteen of them (78.3%, 18/23) established higher cutoff values for IgG than IgM. In four cases (17.4%, 4/23), the cutoff value for IgM was higher, whereas in one case (4.4%, 1/23), identical cutoff values were applied to both isotypes. Ten (12.8%, 10/78) studies targeted both IgG and IgM isotypes. The majority of these studies (50%, 5/10) used a 4-fold or greater increase in titers in paired samples as a diagnostic cutoff. There was a considerable variation in choice of single-titer cutoffs for whole antibody targeting (Table 4).

**Table 4:** Summary of antibody isotype positivity cutoff titer in selected studies

Country	IgG positivity cutoff titer								Studies (*n*)	References
	≥ 1:40	1:64	≥ 1:64	≥ 1:80	≥ 1:128	> 1:128	≥ 1:960	> 1:960		
Brazil		1							1	45
China	1								1	77
Cyprus							1		1	78
France						1			1	40
Germany			1						1	79
Greece			1				1	1	3	48,80,81
Nepal								1	1	82
New Zealand					1				1	39
Spain	1			2	1				4	14,37,58,61
Sri Lanka					1				1	83
Tunisia					1	1			2	19,69
United States		1	2		1				4	84,70,71,85
Total (*n*)	2	2	4	2	5	2	2	2	21	

Country	IgM positivity cutoff titer								Studies (*n*)	References
	≥ 1:32	≥ 1:40	≥ 1:64	> 1:64	≥ 1:80	> 1:80	≥ 1:400	> 1:400		
Brazil				1					1	45
China		1							1	79
Colombia			1						1	88
Cyprus							1		1	78
France				1					1	40
Greece			1				1	2	4	48,80,81,87
Indonesia			1						1	88
Israel		1							1	28
Lao PDR				1			1		2	50,75
Nepal							1		1	16
Spain			1						1	56
Sri Lanka			1						1	83
Taiwan					5	1			6	63,89,90,91,92,93
Tanzania			1						1	65
Tunisia	3		1						4	19,68,67,69
United States			1						1	94
Zambia	1								1	73
Total (*n*)	4	2	8	3	5	1	4	2	29	

Country	Whole antibody positivity cutoff titer								Studies (*n*)	References
	≥ 1:32	≥ 1:50	> 1:50	≥ 1:64	≥ 1:100	≥ 1:200	≥ 1:400	≥ 1:800		
Israel					1				1	74
Lao PDR							1		1	76
Malaysia		1							1	53
Tanzania			1						1	66
Thailand						1		1	2	95
Tunisia	1								1	67
Zambia				1					1	73
Total (*n*)	1	1	1	1	1	1	1	1	8	

* If the positivity cutoff titer was only seen once, then it was not included on the table.

† Studies performed on travelers were excluded.

#### Criteria for selecting cutoffs.

All 78 studies reported at least one positivity criterion. Differentiating by study design, of the 39 case series, a single-titer cutoff was the most commonly used criterion (53.8%, *n* = 21), with the cutoff ranging from ≥ 1:25 to ≥ 1:960 with the majority (17.9%, 7/39) using a titer of ≥ 1:64 (Supplemental Table 3). Four case series (10.3%, 4/39) exclusively used a ≥ 4-fold increase in antibodies in paired samples, whereas 13 (33.3%, 13/39) used this criterion in conjunction with a fixed titer cutoff (Supplemental Table 3). Of the 34 cross-sectional studies, the majority (70.6%, *n* = 24) used a single-titer cutoff to determine positivity, the cutoff ranging from ≥ 1:16 to ≥ 1:4000 with the majority (23.5%, 8/34) using a titer of ≥ 1:64 (Table 1). Only one study (2.9%, 1/34) used exclusively a ≥ 4-fold increase in antibodies as a criterion, whereas eight (23.5%, 8/34) used this criterion together with a fixed titer cutoff (Table 3). Of the five diagnostic accuracy studies, four (80%, 4/5) used a single positivity cutoff titer, ranging from ≥ 1:100 to ≥ 1:400 (Table 2).

Differentiating by country (Table 3), a single-titer cutoff was the preferred method of diagnosis in Cyprus, Greece, Spain, and Tunisia, whereas in Indonesia, Lao PDR, Sri Lanka, Thailand, and United States, a single-titer cutoff in conjunction with a ≥ 4-fold increase in titers was preferred. Only in Taiwan was a solely ≥ 4-fold increase in titers as a diagnostic cutoff preferred.

#### Justification for selecting cutoffs.

Of a total of 78 studies, only 33 (42.3%, 33/78) justified the method to determine their diagnostic cutoff, whereas 45 (57.7%, 45/78) studies provided no clear explanation for the cutoff value used. Of the 33 studies with reasons for their selected cutoff values, 28 (84.8%, 28/33) justified it by citing a supporting previous study. The most frequently cited seropositivity criteria study was that of La Scola et al.^6^ (14.3%, 4/28). Other commonly cited studies were Blacksell et al.,^16^ Coleman et al.,^17^ and Hernandez et al*.*^18^ A further 19 references for justification^19–36^ were cited by 18 studies. Three studies (9.1%, 3/33) used “manufacturers specifications” as a justification for their cutoff values,^37–39^ whereas one study (3.0%, 1/33) followed the “WHO Collaborating Centre procedure” to determine their cutoff.^40^

## DISCUSSION

To classify confirmed cases and to ensure appropriate patient management, the application of accurate diagnostic cutoffs is necessary for murine typhus. This review has found that there was a major lack of consensus regarding methodologies, application, and IFA/IIP positivity cutoffs used for the diagnosis of murine typhus infections; the reasons for which are manifold and need further investigation and standardization.

In many cases (57.7%, 45/78), a clear justification for the cutoff used was not provided, and it is likely that differences in approach evolved naturally based on local antigenic strains and the pretest odds of disease depending on the local level of murine typhus endemicity. This variation raises questions about which, if any, IFA positivity cutoff is most appropriate for the diagnosis of acute murine typhus infection.

Of the five diagnostic accuracy studies, the majority (60%, 3/5) provided sufficient justification for the positivity cutoff titer used. Although there was a lack of consensus in terms of the source used for the reference test, a single positivity cutoff titer of ≥ 1:400 in Lao PDR and ≥ 1:128 in South America was common (Table 2). This is probably an appropriate estimation for certain parts of Lao PDR and South America, with limited application in other geographic locations. As has been previously established, it is also likely that these cutoffs are not befitting for the locations in which they were being used.^41^

La Scola et al.^6^ were most commonly cited as a justification for IFA and IIP diagnostic cutoffs for the diagnosis of *R. typhi.* The study also suggests that although the IFA is an appropriate diagnostic method in the case of acute infections, it should be “considered a technique for seroepidemiology only in areas where the seroprevalence of the rickettsial disease has already been established.” The article emphasizes that the cutoff should be specific for “each rickettsial disease and each area.”^6^

Many studies used identical cutoffs for IgG and IgM (26.9%, 21/78), despite the fact that dynamics of the antibody isotypes differ. This should be considered when interpreting test results, as generally on infection, an increase in IgM is seen, followed by increased levels of IgG.^41,42^

A variety of factors may affect the diagnostic accuracy of IFAs, including the antibody isotype targeted. Differences in IFA single-titer cutoffs were observed in studies where either IgM or IgG were targeted or both IgM and IgG were targeted to apparently increase the accuracy of the test. In general, higher single-titer cutoffs were used for IgG over IgM, whereas no consensus was seen for studies targeting IgM and IgG together (Table 4).

Considering study populations, the use of samples from infected or normal patients and the geographic origin of the patients can influence the consequent diagnostic cutoff. Murine typhus is an important travel-related illness,^7^ and in a few studies, serum samples were collected from various geographic locations, such as Peru, Russia, United States, and Somalia, although there was ambiguity with regard to whether the cutoff was applied to a single population or whether the cutoff was calculated through results from all the populations despite dissimilarities in endemicity. This emphasizes the complexity surrounding murine typhus serology and the lack of consensus. From the data shown here, no single antibody titer can accurately be advised as diagnostic unless preexistent studies have been performed to establish seroprevalence levels in the normal population within a location.

Moreover, in addition to the lack of IFA methodology standardization, there was also a lack of consensus in the reference comparator or “gold standard reference assay” to determine murine typhus diagnostic cutoffs. The absence of standardized methods and validated cutoffs has serious implications for seroepidemiological and clinical research, as well as implications for patients and healthcare workers. Although a lower cutoff would result in increased false-positive results, a higher cutoff would result in increased false-negative results, causing cases to go undiagnosed and increasing the possibility of those patients developing severe complications.

This review has numerous limitations. First, it only investigated studies published in or translated to English. Second, a single author performed the article selection and data extraction, although any ambiguous data were reviewed among the authors to limit bias. Third, the number of diagnostic accuracy studies included was limited, and, perhaps, the study design affects the positivity cutoff titer used for IFA testing. Therefore, it is difficult to conclude whether there exists a correlation or causation between study designs and cutoff titers. Fourth, this review did not consider the timing of serum collection and the collection of paired sera in relation to the disease. The timing of sample collection in relation to illness onset is an important factor to consider when analyzing a positive serological result. Last, the IFA protocol was not assessed as a factor. This is essential to consider when analyzing results, as variances in protocol (i.e., the quantity of antigen used and inactivation techniques) can affect the sensitivity and specificity of IFA tests, which in turn can affect the selection of optimal cutoffs. Moreover, this review examined both IFA and IIP tests, and the two protocols were not differentiated in this study.

From this review, we cannot conclude a single standardized cutoff titer for murine typhus; however, there are some clinical aspects that are important to note. In terms of treatment, murine typhus is treatable with doxycycline,^43^ which is an affordable and safe drug. It is possible to prescribe doxycycline in patients who present with non-malarial febrile illness symptoms; however, it could result in no effect as the patient may be infected with a disease not sensitive to doxycycline. Thus, it is essential to accurately diagnose the disease in patients, for which a validated threshold is needed. In highly endemic areas, there are high backgrounds of murine typhus, which poses a potential for false positivity if the cutoff is set too low.

Further research is required to examine the local levels of background immunity, identify circulating antigenic strains, and assess different IFA testing protocols, to make well-versed decisions regarding a region-specific, standardized IFA methodology and cutoff. The prospective cause of fever studies could be carried out in different geographical localities in urban versus rural areas to validate an optimal region-specific cutoff. Moreover, the timing of serum collection and pairing of sera could be assessed to formulate a criterion to classify confirmed versus probable cases, rather than focus on a single-titer cutoff.

## Supplemental tables

Supplemental materials
