# Supplementary material for: Selection of Diagnostic Cutoffs for Murine Typhus IgM and IgG Immunofluorescence Assay: A Systematic Review
Source: Am J Trop Med Hyg. 2020 Apr 6;103(1):55–63. doi: 10.4269/ajtmh.19-0818 (PMC7356422; doi:10.4269/ajtmh.19-0818)
Supplement: Supplementary file 1 [file tpmd190818.SD1.pdf]

PRISMA 2009 Flow Diagram

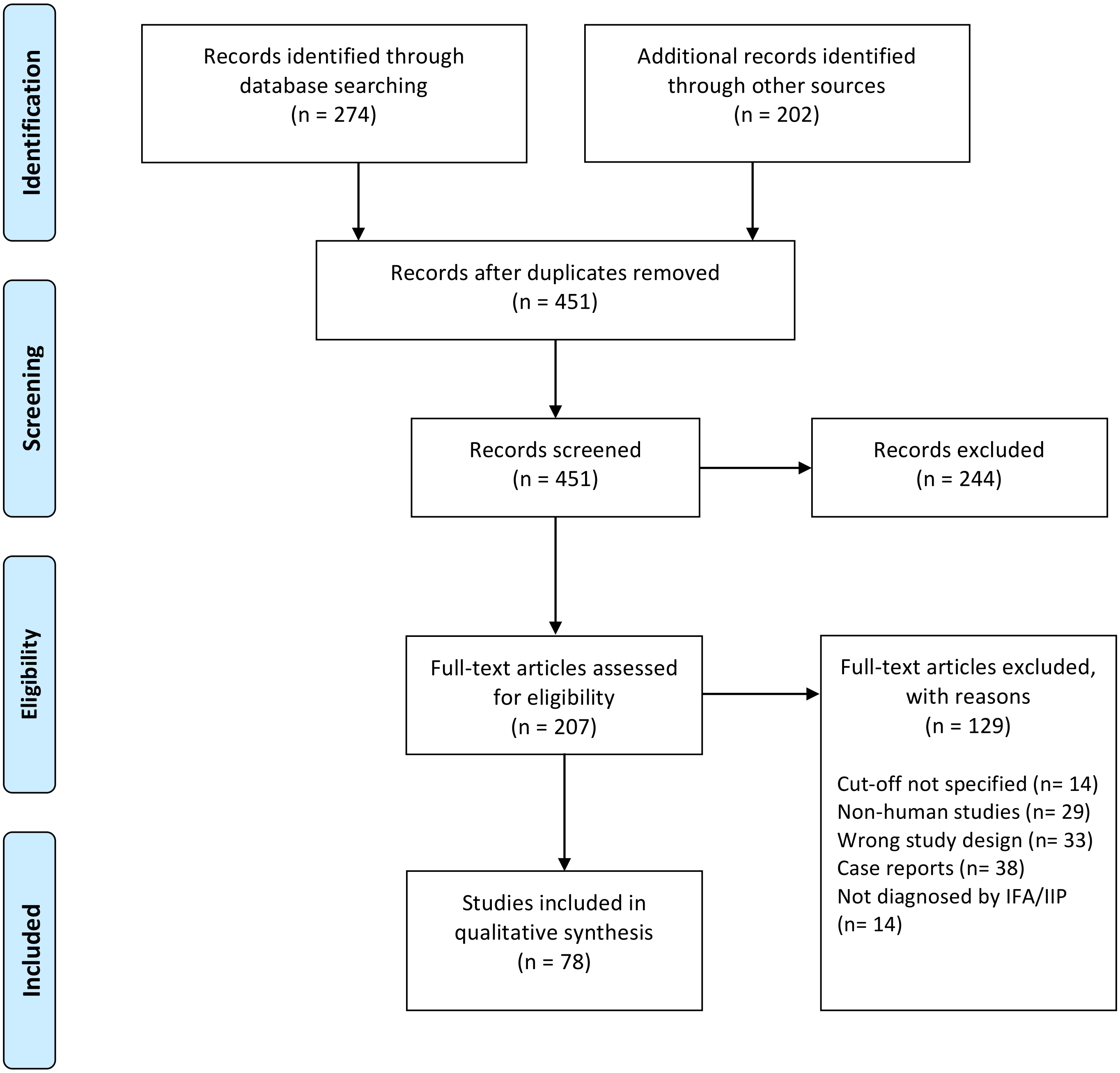

From: Moher D, Liberati A, Tetzlaff J, Altman DG, The PRISMA Group (2009). *P*referred *R*eporting *I*tems for *S*ystematic Reviews and *M*eta-*A*nalyses: The PRISMA Statement. PLoS Med 6(7): e1000097. doi:10.1371/journal.pmed1000097

For more information, visit www.prisma-statement.org.
