# Supplementary material for: Selection of Diagnostic Cutoffs for Murine Typhus IgM and IgG Immunofluorescence Assay: A Systematic Review
Source: Am J Trop Med Hyg. 2020 Apr 6;103(1):55–63. doi: 10.4269/ajtmh.19-0818 (PMC7356422; doi:10.4269/ajtmh.19-0818)
Supplement: Supplementary file 3 [file tpmd190818.SD3.docx]

# S3: Summary of IFA case series

| **Country** | **Type of Test** | **Source of assay** | **Total Cases** | **Antigenic Strain** | **Positivity cut-off titer** | **Antibody Target** | **Positivity Criteria** | **Cut-off Justification** | **Reference** |
| --- | --- | --- | --- | --- | --- | --- | --- | --- | --- |
| **Australia** | IFA | In-house | 131 | NA | 1:128 | IgG | Single titre | NA | ^97^ |
| **African and South East Asian travellers** | IFA | NA | 32 | NA | ≥ 1:64 | IgM | Single titre | ^19^ | ^12^ |
|  |  |  |  |  | ≥ 1:128 | IgG | Single titre |  |  |
|  |  |  |  |  | ≥ 4-fold increase | IgM and IgG | Only 4-fold |  |  |
| **China** | IFA | NA | 76 | Wilmington | ≥ 1:40 | IgM | Single titre | NA | ^78^ |
|  |  |  |  |  | ≥ 1:40 | IgG | Single titre |  |  |
| **Colombia** | IFA | NA | 120 | Wilmington | ≥ 4-fold increase | IgG | Only 4-fold | NA | ^87^ |
|  |  |  |  |  | ≥ 1:64 | IgM | Single titre |  |  |
| **Croatia** | IFA | Biomerieux, Marcy l'Etoile, France | 126 | NA | ≥ 4-fold increase | Whole | Only 4-fold | ^6^, ^29^ | ^98^ |
| **Cyprus** | IFA | NA | 21 | NA | ≥ 1:960 | IgG | Single titre | NA | ^79^ |
|  |  |  |  |  | ≥ 1:400 | IgM | Single titre |  |  |
|  | IFA | Biomerieux, Marcy l'Etoile, France | 193 | NA | ≥ 1:480 | IgG | Single titre | NA | ^99^ |
|  |  |  |  |  | ≥ 1:200 | IgM | Single titre |  |  |
| **France** | IFA | In-house | 299 | NA | ≥ 1:25 | NA | Single titre | ^6^, ^30^ | ^100^ |
|  | IFA | NA | 8 | NA | > 1:64 | IgM | Single titre | WHO Collaborating Centre procedure | ^40^ |
|  |  |  |  |  | > 1:128 | IgG | Single titre |  |  |
| **Germany** | IFA | In-house | 28 | Wilmington | ≥ 1:64 | IgG | Single titre | NA | ^80^ |
| **Greece** | IFA | Biomerieux, Marcy l'Etoile, France | 41 | NA | > 1:400 | IgM | Single titre | NA | ^88^ |
|  | IFA | NA | 49 | NA | 1:160 (if patient had fever) | NA | Both | Tested antibody levels of 30 asymptomatic factory workers, and derived a mean to determine cut off value (explained in methods) | ^24^ |
|  |  |  |  |  | 1:1280 (if patient did not have fever) | NA |  |  |  |
|  |  |  |  |  | ≥ 4-fold increase | NA |  |  |  |
|  | IFA | NA | 90 | NA | ≥ 1:960 | IgG | Single titre | NA | ^81^ |
|  |  |  |  |  | ≥ 1:400 | IgM | Both |  |  |
|  |  |  |  |  | ≥ 4-fold increase | IgM |  |  |  |
|  | IFA | Biomerieux, Marcy l'Etoile, France | 49 | NA | > 1:400 | IgM | Single titre | ^23^, ^24^ | ^82^ |
|  |  |  |  |  | > 1:960 | IgG | Single titre |  |  |
| **Indonesia** | IFA | Biomerieux, Marcy l'Etoile, France | 137 | NA | ≥ 1:64 | IgM | Both | NA | ^89^ |
|  |  |  |  |  | ≥ 1:256 | IgG |  |  |  |
|  |  |  |  |  | ≥ 4-fold increase | IgM and IgG |  |  |  |
| **Israel** | IFA | NA | 52 | NA | ≥ 1:40 | IgM | Single titre | ^101^ | ^28^ |
| **Lao PDR** | IFA | Australian Rickettsial Reference Lab | 248 | Wilmington | ≥ 4-fold increase | Whole | Only 4-fold | ^55^ | ^102^ |
|  | IFA | Australian Rickettsial Reference Lab | 248 | Wilmington | ≥ 4-fold increase | Whole | Only 4-fold | ^55^ | ^103^ |
| **Malta** | IFA | NA | 33 | Wilmington | 1:64 | IgM | Single titre | NA | ^104^ |
| **Nepal** | IFA | Australian Rickettsial Reference Lab | 627 | NA | > 1:960 | IgG | Single titre | NA | ^83^ |
|  |  |  |  |  | ≥ 4-fold increase | IgM | Only 4-fold |  |  |
| **Sri Lanka** | IFA | NA | 20 (first batch) | Wilmington | > 1:20 – > 1:5120 | IgM | Range | ^6^ | ^105^ |
|  |  |  | 40 (second batch) |  | > 1:50 | IgM | Single titre |  |  |
|  | IFA | NA | 178 | NA | ≥ 1:64 | IgM | Both | ^27^ | ^84^ |
|  |  |  |  |  | ≥ 1:128 | IgG |  |  |  |
|  |  |  |  |  | ≥ 4-fold increase | IgM and IgG |  |  |  |
| **Taiwan** | IFA | NA | 81 | NA | ≥ 1:80 | IgM | Single titre | ^18^ | ^90^ |
|  |  |  |  |  | ≥ 4-fold increase | IgG | Only 4-fold |  |  |
|  | IFA | NA | 441 | NA | ≥ 1:80 | IgM | Single titre | ^18^ | ^91^ |
|  |  |  |  |  | ≥ 4-fold increase | IgG | Only 4-fold |  |  |
|  | IFA | NA | 856 | NA | ≥ 1:80 | IgM | Single titre | ^18^ | ^92^ |
|  |  |  |  |  | ≥ 4-fold increase | IgG | Only 4-fold |  |  |
|  | IFA | Taiwan CDC | 106 | NA | > 1:80 | IgM | Single titre | NA | ^93^ |
|  |  |  |  |  | ≥ 4-fold increase | IgG | Only 4-fold |  |  |
|  | IFA | Taiwan CDC | 413 | NA | ≥ 1:80 | IgM | Single titre | NA | ^94^ |
|  |  |  |  |  | ≥ 4-fold increase | IgG | Only 4-fold |  |  |
| **Thailand** | IFA | NA | 94 | Wilmington | ≥ 1:400 | NA | Both | ^22^ | ^106^ |
|  |  |  |  |  | ≥ 4-fold increase | NA |  |  |  |
|  | IFA | US Army Medical Research Unit-Malaysia | 19 | Wilmington | ≥ 1:400 | NA | Both | ^33^ | ^22^ |
|  |  |  |  |  | ≥ 4-fold increase | NA |  |  |  |
|  | IIP | US Army Medical Research Unit-Malaysia | 37 | NA | ≥ 1:200 | Whole | Both | NA | ^96^ |
|  |  |  |  |  | ≥ 4-fold increase | Whole |  |  |  |
|  |  |  |  |  | ≥ 1:800 | Whole |  |  |  |
|  | IIP | US Army Medical Research Unit-Malaysia | 137 | Wilmington | ≥ 4-fold increase | NA | Both | ^22^, US Army Medical Research Unit Manual, 1985 | ^107^ |
|  |  |  |  |  | ≥ 1:400 | NA |  |  |  |
|  | IIP | US Army Medical Research Unit-Malaysia | 320 | NA | ≥ 1:400 | NA | Both | ^33^, US Army Medical Research Unit Manual, 1985 | ^108^ |
|  |  |  |  |  | ≥ 4-fold increase | NA |  |  |  |
| **Tunisia** | IFA | NA | 73 | NA | 1:128 | IgM | Single titre | NA | ^109^ |
|  | IFA | NA | 280 | NA | ≥ 1:64 | IgM | Both | NA | ^19^ |
|  |  |  |  |  | ≥ 4-fold increase | IgM and IgG |  |  |  |
|  |  |  |  |  | ≥ 1:128 | IgG |  |  |  |
|  | IFA | NA | 7 | NA | ≥ 1:512 | NA | Single titre | NA | ^110^ |
| **USA** | IFA | NA | 97 | NA | ≥ 1:128 | NA | Both | NA | ^111^ |
|  |  |  |  |  | ≥ 4-fold increase | NA |  |  |  |
|  | IFA | NA | 1,135 | NA | ≥ 1:64 | IgM | Both | NA | ^95^ |
|  |  |  |  |  | ≥ 1:128 | NA |  |  |  |
|  |  |  |  |  | ≥ 4-fold increase | NA |  |  |  |
|  | IFA | NA | 345 | NA | ≥ 1:128 | NA | Both | NA | ^86^ |
|  |  |  |  |  | ≥ 4-fold increase | NA |  |  |  |
|  | IFA | NA | 18 | NA | 1:64 | IgG | Both | NA | ^85^ |
|  |  |  |  |  | ≥ 4-fold increase | IgG |  |  |  |
